# Multiple sensor theory in cardiovascular mechanosensory units

**DOI:** 10.3389/fphys.2022.1044577

**Published:** 2023-01-17

**Authors:** Jerry Yu

**Affiliations:** ^1^ Department of Medicine, University of Louisville, Louisville, KY, United States; ^2^ Robley Rex VA Medical Center, Louisville, KY, United States

**Keywords:** baroreceptor, atrial receptor, baroreflex, vagal afferent, mechanoreceptor, cardiovascular reflex

## Abstract

Multiple sensor theory (MST) has advanced our understanding of how lung mechanosensors operate. That is, single lung units contain multiple homogeneous or heterogeneous sensors. Each detects sensor-specific mechanical information and interacts with other sensors lying within the unit sending integrated information to the brain to evoke reflexes. MST explains numerous controversial issues in the respiratory system. Recent studies in baroreceptors (BRs), along with reinterpretation of recordings appearing in the literature, indicate MST also operates in the cardiovascular (CV) system. This review outlines evidence supporting MST in the CV system and provides examples to apply the theory. Longstanding controversies surrounding the CV sensors are also considered.

## Introduction

The walls of the heart, large arteries and veins house mechanical and chemical sensors that continuously monitor hemodynamic events in the circulatory system. This information is carried to the central nervous system (CNS) in both vagal and sympathetic afferents to initiate reflexes. Mechanical information is mainly carried in the vagus nerves in mechanosensors sensitive to stretch. Pulsatile distortion during each heart beat generates action potentials (APs) that provide beat-to-beat regulation of hemodynamics. These sensory units have been explored for almost a century, yet their fundamental operating mechanisms remain unclear, largely due to misconception, such as one sensor theory (OST) and line-labeled theory. In OST, one mechanosensor connects to a single afferent axon. In line-labeled theory, different sensor types project to different CNS areas to evoke specific responses. There are limitations to these approaches, however. In the respiratory system, two types of mechanosensors are posited: rapidly adapting receptors (RARs) stimulate inspiration (excitatory line) and slowly adapting receptors (SARs) inhibit inspiration (inhibitory line). However, a series of recent studies demonstrate that RARs and SARs may share a single axon to form a sensory unit, transmitting different signals to the CNS (Yu, 2016). These results challenge line-labeled theory and make OST no longer tenable. Under multiple sensor theory (MST) many heterogeneous mechanosensors operate in a single unit (Yu, 2020). Each has its own characteristics of activation and deactivation thresholds, saturation pressure, adaptation rate, operating range, discharge variability, and sensory mode (rate and magnitude; inflation and deflation). Different sensor combinations give varied behaviors (Yu, 2005). At any instant, the highest discharging sensor serves as the pacemaker and determines unit discharge frequency. The final discharge pattern depends on the composition of sensors and their interaction. A recent aortic baroreceptor (BR) study demonstrates multiple sensors also operate in BR units (Liu et al., 2021). Careful literature review points to MST as a common sensory mechanism applicable to the cardiovascular (CV) system. This article provides an overview of vagal CV mechanosensory units, focusing on structure-function integration to illustrate MST.

### General electrophysiology

Located in strategic sites, CV mechanosensors send signals to the CNS *via* nodose and petrosal ganglia. Units lying in low-pressure sites (endocardium, vena cava and pulmonary artery) show similar structural and physiological features to those in high-pressure sites (aortic, carotid, and coronary arteries). They are also similar to those in the airway and allow comparative evaluation (Yu, 2022). Since morphologic Larsell and Dow (1933) and physiologic studies show there are fewer CV sensors in the pulmonary artery (Coleridge and Kidd, 1960), we will focus on aortic, carotid and atrial sensors.

**FIGURE 1:**
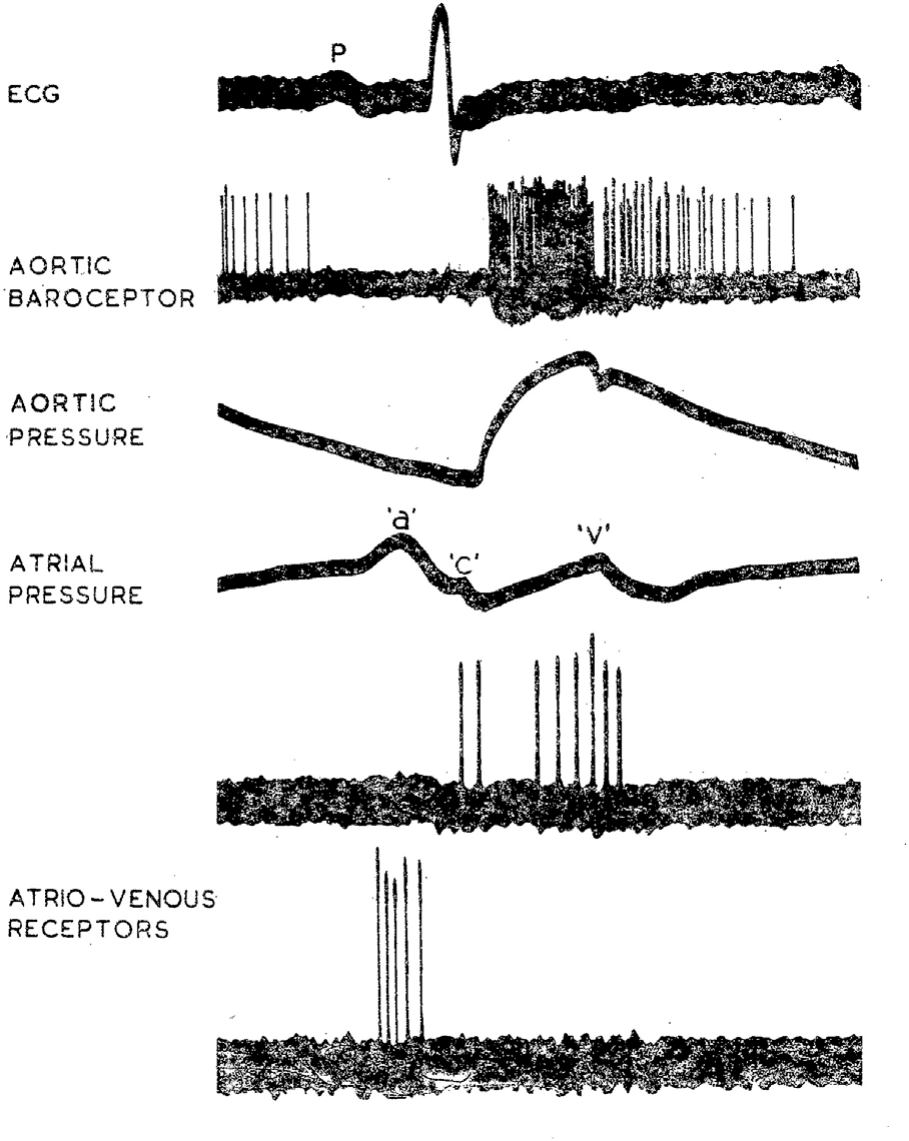
Characteristic discharge patterns in relation to ECG and pressure pulse in myelinated vagal “high-pressure” and “low-pressure” mechanoreceptors in the dog. The aortic BR was near the origin of the left subclavian artery. Activity in the upper of the two atrio-venous recordings arose from an ending (type B) at the junction of right atrium and superior vena cava; activity in the lower trace came from an ending (type A) in the superior vena cava just inside the pericardium. [Figure 1 (Coleridge et al., 1978)].

CV mechanosensors are innervated by thick myelinated afferents that generate high discharge frequencies of 200–300 impulses/sec synchronized with CV events (Coleridge et al., 1981). Thus, they are able to detect small differences in pressure or dp/dt. Such timed characteristic discharge permits investigators to locate sensor sites. For example, arterial BR discharge synchronizes with rising aortic pressure pulses and atrial receptors discharge in phase with atrial contraction (a wave) and filling (v wave), respectively (Figure 1). Location of these different sensors can be distinguished by manipulating distending pressure in various sites (Coleridge et al., 1957).

Different types of BRs are found. For example, carotid BRs can be classified into type I and type II. Type I has high discharge frequency and sensitivity, whereas type II has low firing frequency and sensitivity (Brown et al., 1976). Type I sensors connect to large A fibers and type II to small A or C fibers (Seagard et al., 1990). Type I and II BRs are believed to provide acute and chronic regulation of BP, respectively (Seagard et al., 1993). In general, large myelinated arterial BRs are believed to slowly adapt because at a pressure step the activity increases, followed by an initial rapid and then slow decline over time (Chapleau et al., 1991; Koushanpour, 1991). The initial component is believed to result from mechanical effects of the vessel.

### General morphology

**FIGURE 2:**
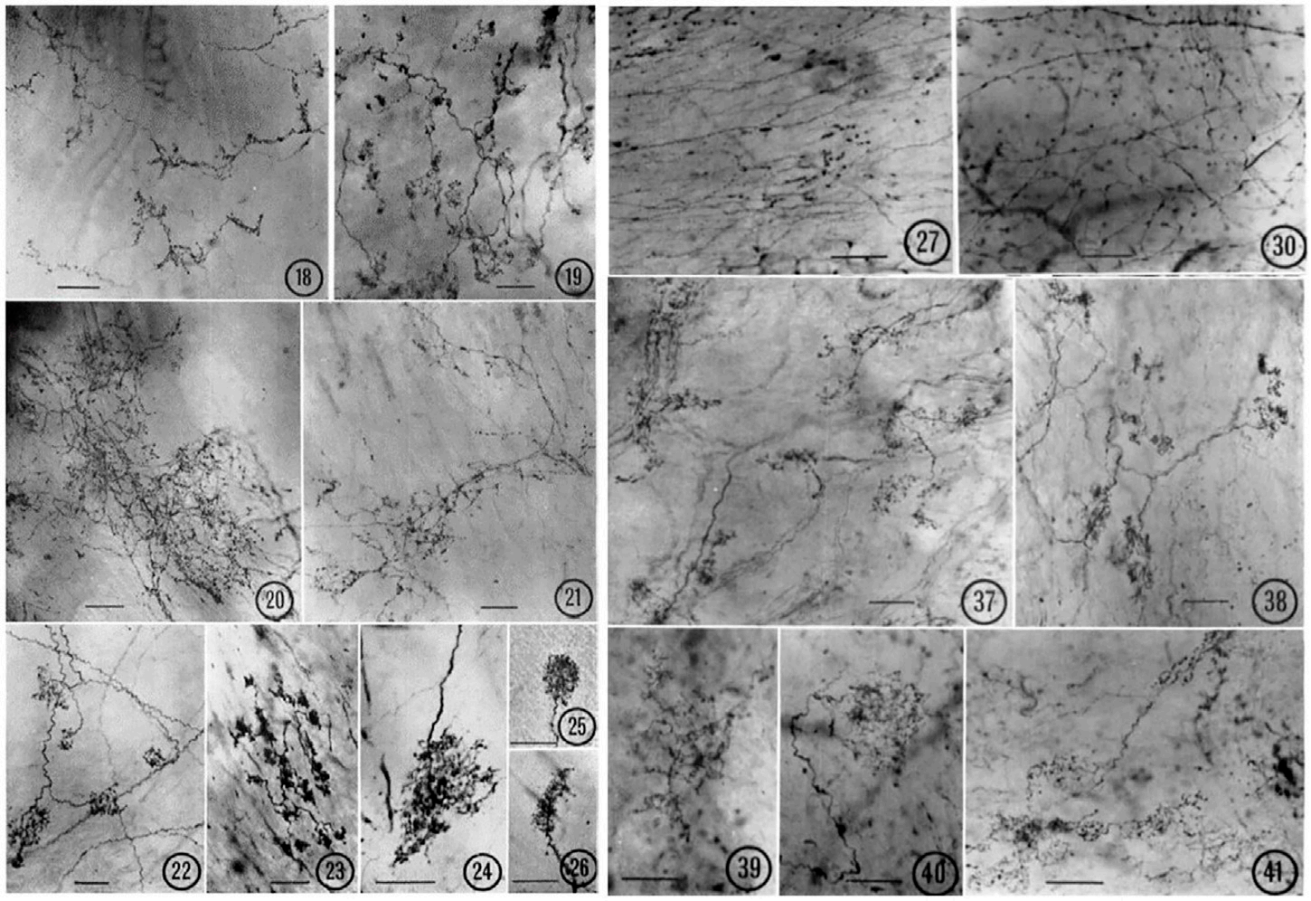
Various types of atrial receptors. 18–21 are diffuse CUEs. 18, cat left atrial endocardium (tending toward compact, intermediate type); 19, cat right atrial endocardium; 20, lamb right atrial endocardium; 21, monkey left atrial endocardium. 22–26 are compact CUEs. 22, dog left atrial endocardium; 23, monkey left atrial endocardium; 24, lamb left atrial endocardium; 25, cat right atrial endocardium; 26, cat left atrial endocardium. Sensory end-nets in the endocardium of right interventricular septum (27, cat) and in the mitral valve (30, Monkey). CUEs in the adventitia of the aortic arch (37, cat; 38, lamb; 39, monkey; 40–41, Dog). [Figures (Miller and Kasahara, 1964)].

Two basic types of end-formations are described in CV sensors: complex unencapsulated endings (CUEs) and end-nets. Early studies exploring sensory structures used silver impregnation (Ag) to examine 15 *μ* sections; methylene blue to stain whole mount; and osmium tetroxide to detect myelinated fibers. In general, vagal sensory structures are identified by their survival (no degeneration) after vagotomy. These structures are largely preserved and similar in different sensory locations across species (Figure 2). For example, similar CV receptors are found in the atrium, ventricle, arteries (aortic, carotid, pulmonary), and pulmonary veins across species (human, pig, cow, dog, and cat) (Abraham, 1969).

Various end-nets and CUEs have been described (Miller and Kasahara, 1964) (Figure 2). End-nets are formed by the anastomoses of several branches of fibers. Because of the quality of early figure production, limitations of microscopy, and differences in staining techniques, it is difficult to determine sensory structures definitively, especially the end-net type. While the origin of end-nets is debatable, they are generally believed to be unmyelinated nerve fibers (Johnston, 1968; Hainsworth, 1991a). In the late 1970 s, with confocal microscopy and neural tracer techniques, end-net and flower-spray (CUE) endings were often found colocalized in the endocardium near the entrance of major vessels in both atria (Cheng et al., 1997). This indicates that at least some net-nets are vagal afferent structures.

**FIGURE 3:**
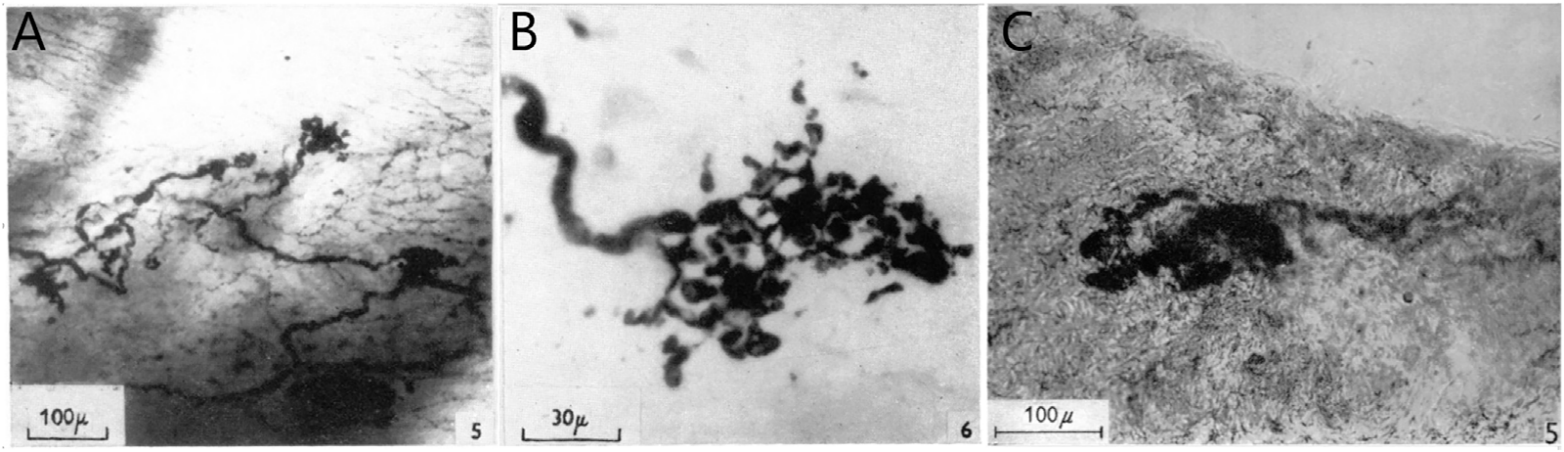
**(A)**, Thick nerve fiber terminal expansions in the posterior wall of the left atrium. In the right background, fine plexiform terminal network (end-net) can be seen. Whole mount methylene blue preparation. **(B)**, high-power photomicrograph of a typical compact CUE (flower-spray). [Figure 5 and Figure 6 (Coleridge et al., 1957)]. **(C)**, portion of tunica media showing diffuse positive reaction for non-specific cholinesterase. This contains a small area in which the reaction is strongly positive, revealing a structure similar to those displayed by the silver technique. Modified Koelle procedure for non-specific cholinesterase. [Figure 5F (Coleridge et al., 1961)].

CUEs can be divided into diffuse and compact types (Figure 2). The diffuse types have moderate sized myelinated fibers (4–6 μm) with many branches that occupy a considerable area covering several tenths of a square mm. Compact types are more spatially circumscribed (50–350 µm in size) and number about 200 in the dog endocardium (Miller and Kasahara, 1964). They have moderate to large myelinated axon fibers (8–14 μm). Some intermediate forms are found, underscoring the artificiality of this or any other classification. Atrial receptor morphology has been described in detail with ample illustrations (Floyd, 1979). Similarly, detailed account of diffuse and compact CUEs on BRs was described as delicate rings or as small club-shaped dilations (Nonidez, 1935). The morphologies of aortic and carotid BRs, both diffuse and compact, are strikingly similar (Aumonier, 1972). Using electrophysiologic recording to identify the receptive field, both atrial (Coleridge et al., 1957) and pulmonary (Coleridge et al., 1961) mechanosensors are myelinated compact CUEs (Figure 3). They are similar to those identified in cat aortic BRs [Figure 5 of Ref (Aumonier, 1972)] and in airway SARs (Yu et al., 2003; Yu et al., 2004).

### Dilemmas associated with one sensor theory

**FIGURE 4:**
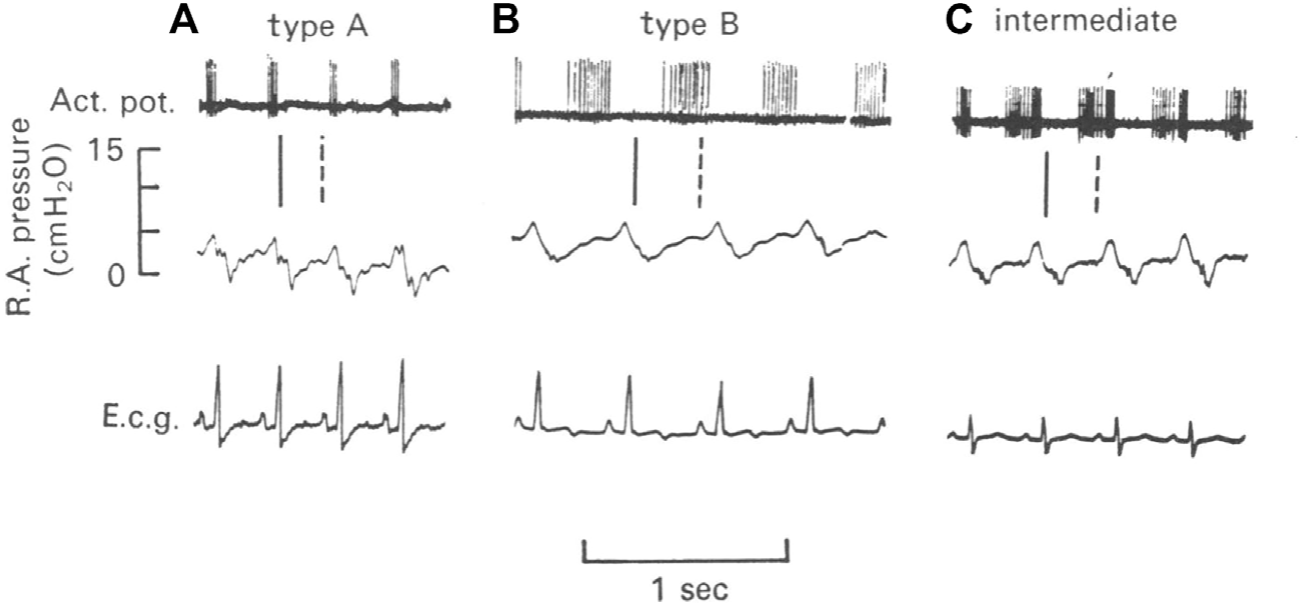
Illustration of three types of atrial units in the dog. **(A)**, type A unit. **(B)**, type B unit. **(C)**, an intermediate unit. Top, action potentials; Middle, right atrial pressure; and Bottom, electrocardiogram (e.c.g.). The vertical lines denote the temporal relationship between action potentials and the atrial pressure waves. The continuous line is the end of the “a” wave and the interrupted line is the peak of the “v” wave (Kappagoda et al., 1977)

Two types of atrial receptors were first described on the basis of discharge patterns related to atrial pressure waves (Paintal, 1953). Type A discharges at high frequency during the atrial ‘‘a’’ wave to signal atrial contraction, whereas Type B discharges at low frequency during the ‘‘v’’ wave to detect the stretch of atrial filling (Figure 1). Later a third intermediate type (A-B type) showed both ‘‘a’’ and ‘‘v’’ bursts (Paintal, 1963; Paintal, 1979) (Figure 4).

**FIGURE 5:**
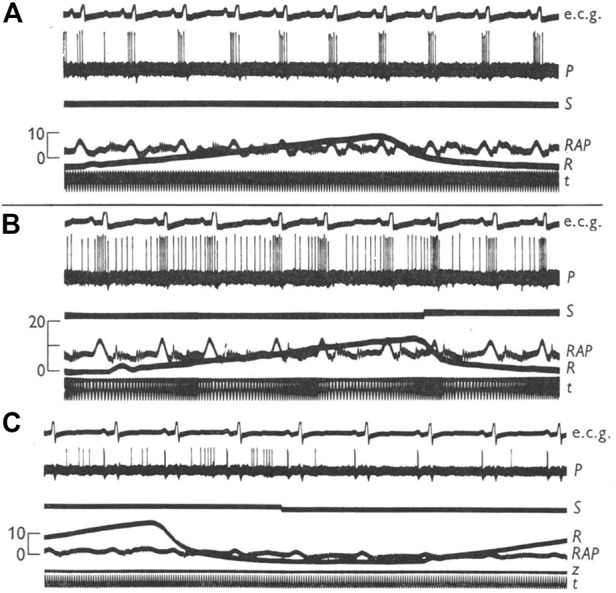
**(A)**, a type A right atrial unit showing a volley of impulses synchronous with the “a” wave in the atrial pressure record; **(B)**, infusion of 200 ml normal saline into the femoral vein (signal marker indicates the end of the infusion) brought out a late systolic volley. **(C)**, another type A unit, showing occlusion of inferior vena cava does not affect the high frequency “a” wave discharge (marker indicates onset of occlusion). Ecg, electrocardiogram; *P*, unit impulses; S, signal marker; RAP, right atrial pressure; R, airway pressure; t, time marker. [Figure 2 and Figure 4 (Coleridge et al., 1957)].

**FIGURE 6:**
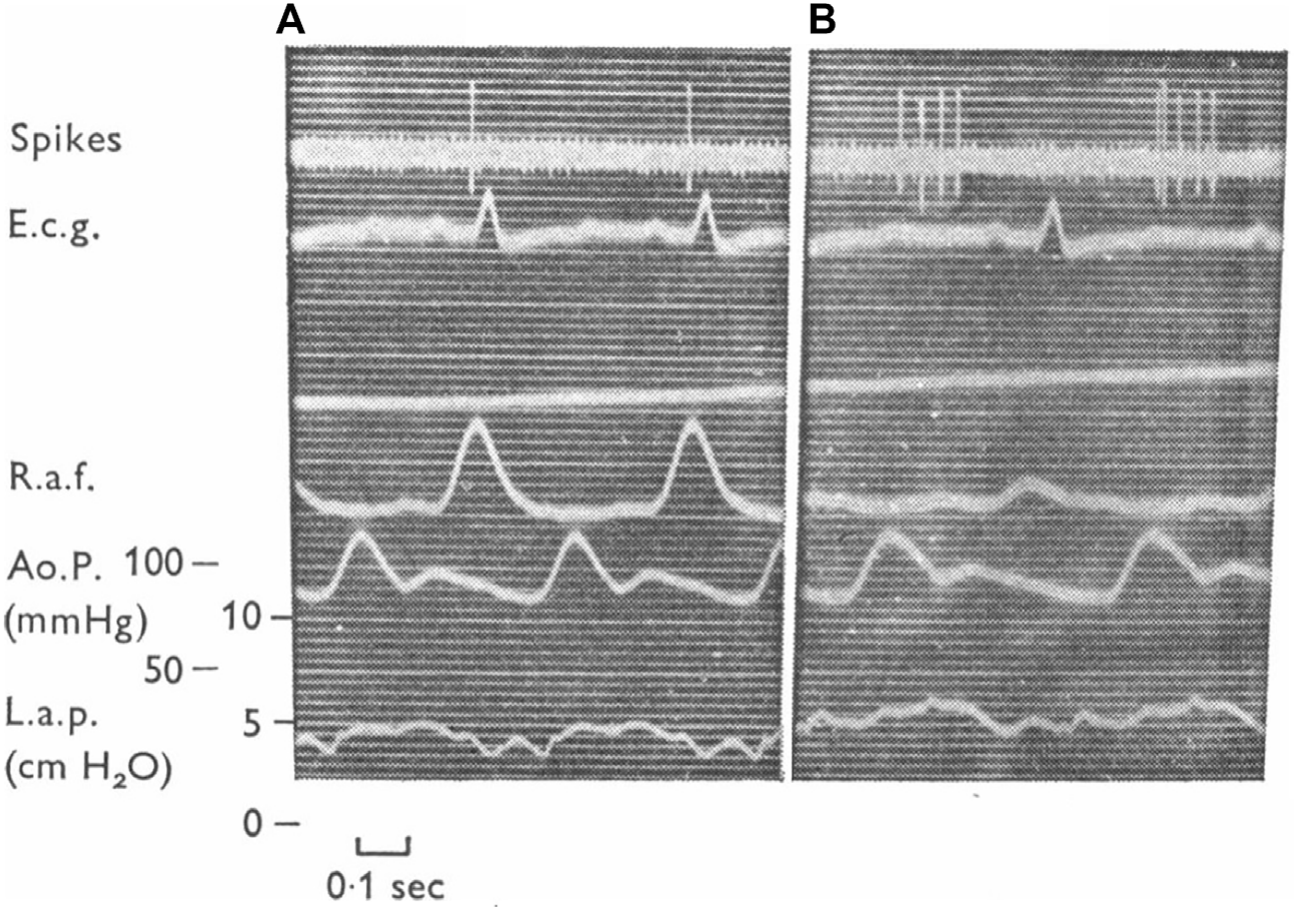
A left atrial unit before **(A)** and after **(B)** administration of propranolol. Abbreviations from top down are unit activity, EKG, right atrial force, aortic pressure and left atrial pressure. This unit exhibits type **(A)** activity during the control and type **(B)** following propranolol. In the opinion of these authors the receptors with **(A)**- and **(B)**-type firing constitute a rather homogeneous group of endings and their different discharge patterns are related mainly to their location in the atria. [Figure 12 (Gilmore and Zucker, 1974)].

However, infusion of saline into the femoral vein was found to produce v wave discharge (i.e., “v” burst) in some type A units, without significant effects on “a” burst (Coleridge et al., 1957). Conversely, occlusion of the inferior vena cava could remove intermediate unit-associated “v” burst without affecting the “a” burst (Figure 5). Similarly, an A type unit may convert to a B type after administration of propranolol (Gilmore and Zucker, 1974) (Figure 6). Important questions emerge. How many types of atrial receptors are there? Are Types A and B the same or different? What are their functions?

Under OST, inter-conversion between A and B types indicates they are the same receptor presenting different behaviors under different states. Detailed studies in cats, dogs and rabbits show increasing atrial pressure or volume can convert Type A to intermediate or Type B, while decreasing them converts Type B to intermediate or Type A (Kappagoda et al., 1976; Kappagoda et al., 1977). These conversions can be induced by different hemodynamic interventions such as infusion of fluids, bleeding, pressor agents, circulatory occlusion, or respiratory maneuvers. Thus, different type units may be a homogeneous population. Additional support rests with no morphologic differences between physiologically recorded Type A and B. Both are flower-spray (Coleridge et al., 1957). Types A and B behave the same after being isolated and tested under sinusoidal stretch (Arndt et al., 1974). Accordingly, their physiologic differences *in situ* may be due to anatomic location and not their basic properties, e.g., Type As lie in the atria, while Type Bs lie near the veins. However, others report no differences in location, with both concentrated at the junctions of the veins in the atrium (right and left), suggesting differences resulting from their arrangements within contractile elements in the atrial wall (Gupta, 1977a; Gupta, 1977b).

Arndt believed that Type A activity increases with increasing heart rate and inotropic state but not atrial volume changes. On the other hand, Type B is sensitive to changes in central blood volume proportional to wall strain. That is, different information is conveyed by A and B receptors, although their behavior is the same *in vitro* (Arndt, 1979). Similarly, A and B are maintained to be truly different types; however, intermediate types can be regarded as extreme variations of type A and B receptors (Paintal, 1979). It should be emphasized that some sensors never convert: they stay the same regardless of hemodynamic manipulations. Paintal considered the possibility of two heterogeneous sensors (types A and B) connecting to the same afferent fiber, but dismissed it on the grounds of contradiction to Muller’s law of specific energies (Paintal, 1979). That is, if multiple sensors do exist in a single unit, these sensors have to be homogeneous. These sensor type issues have been extensively reviewed (Arndt, 1979; Hainsworth, 1991a; Hainsworth, 1991b; Schultz, 2005; Bishop et al., 2011), but the problem remains unresolved.

**FIGURE 7:**
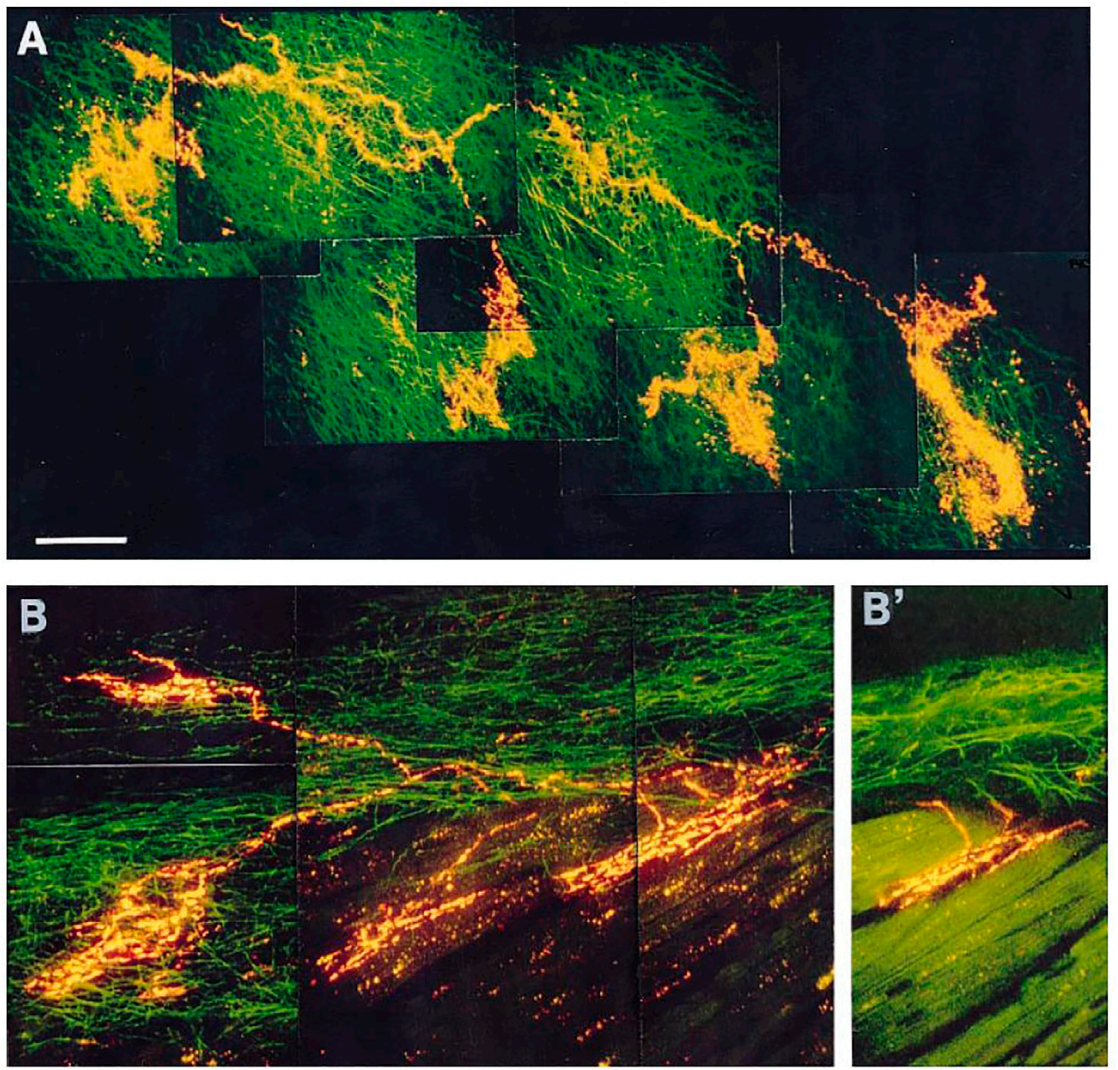
**(A)**: Receptive field of a vagal afferent. A single afferent fiber from the top left, covering a large receptive zone, makes four flower-sprays in the connective tissues of endocardium. **(B)**: Polymorphic receptive field. A single afferent fiber produces two intramuscular endings (IMEs, on the right) and two flower-sprays (on the left). **(B)’**: An optical section from panel B indicating that the IMEs are parallel-elongated. [Figure 4 (Cheng et al., 1997)].

Using a DiI-labeled nodose atrial preparation, heterogeneous sensor types (intramuscular and flower-spray endings) may connect to a single axon (Figure 7). Under OST, intramuscular endings may be Type A to sense muscle contraction; flower-spray endings may be Type B to monitor atrial volume; with polymorphic endings, Type A-B, showing both a and v bursts (Cheng et al., 1997). Such correlations can explain the existence of the three types of sensors, but not the conversion of firing patterns. In a recent study, Type Bs were found not to operate at room temperature while Type As do. The two types were proposed to operate under different molecular transduction mechanisms (Campbell et al., 2020). Again, discharge pattern conversion is puzzling and usually not accompanied by temperature changes. Clearly, interpretation based on OST is problematic.

### Evidence of multiple sensor theory

MST provides an alternative interpretation. In Figure 7, a parent axon connects to four sensory end-formations (homogeneous structures in A and heterogeneous in B). Questions arise as to where APs are generated. At each end-formation? Or at a point farther up in the parent axon? According to OST, each active end-formation is a contributor to AP generation, i.e., it is only part of the sensor or unit. Accordingly, a unit is merely a transducer. Its sensory endings transform mechanical energy into electrical energy and generate APs to the CNS to initiate reflex effects. In MST, each end-formation is a sensor that can generate APs by itself. The final discharge pattern of the parent axon results from the interaction of APs generated from homogeneous (Figure 7A) or heterogeneous (Figure 7B) sensors. Each sensor performs signal transduction and encodes information in the form of APs that interact with others in the unit to form the final train of APs (integration). This integrated information is sent to CNS for further information processing. Thus, the unit is not only a transducer but also an integrator (Yu, 2005). This concept significantly shifts the conventional view. The sensor itself does not exhibit polymorphism. Rather, the unit represents polymorphism, meaning it contains heterogeneous sensors.

**FIGURE 8:**
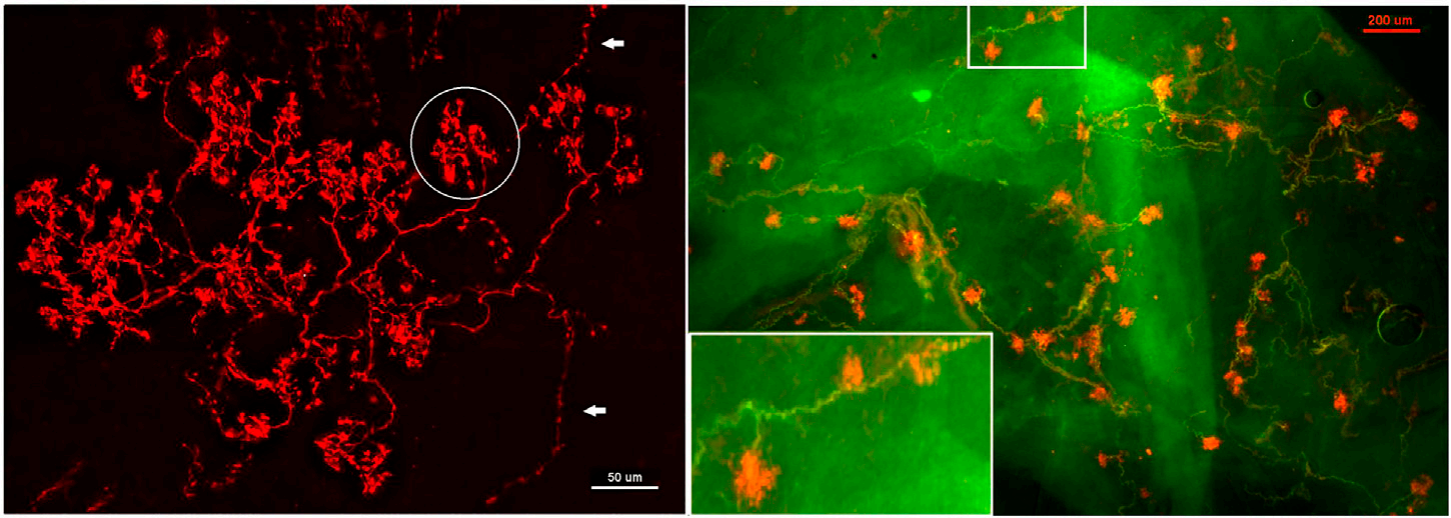
Aortic baroreceptor structures. Left: sensory structures in compact clusters resembling bunches of grapes. Their terminals swell to form knob-like or leaf-like end-formations. A single parent axon may connect with multiple sensory receptor structures, indicated by white arrows. Circled in white is a single BR (a compact CUE). The scale bar is 50 μm. Right: a double staining approach to illustrate BR structures. Na^+^/K^+^ -ATPase stains all structures in the sensory unit (red) and myelin basic protein (MBP) stains the myelin sheath (green). Each red ball shaped end-formation (a compact CUE) is a BR, demonstrated by a myelinated axon termination. The lower left corner is an enlargement of the structure in the upper middle rectangle. The scale bar is 200 μm [Figure 1 and Figure 2 (Liu et al., 2021)].

**FIGURE 9:**
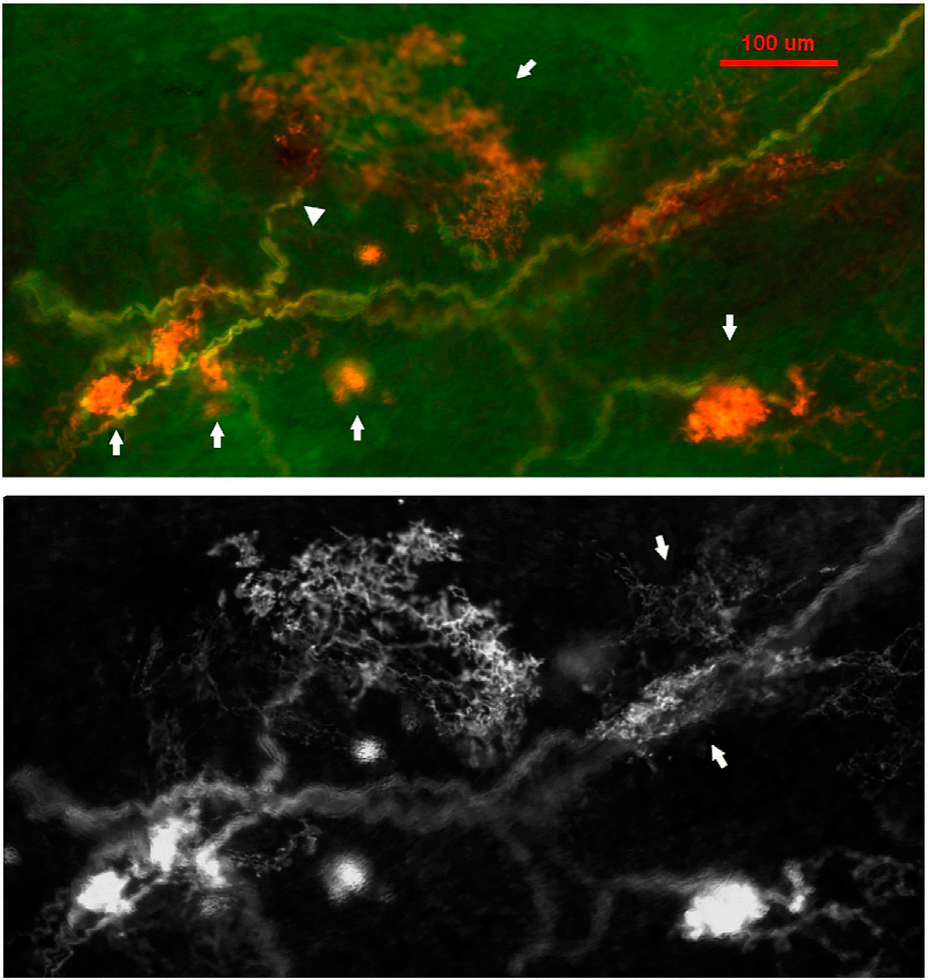
A double staining approach (Na^+^/K^+^ -ATPase and MBP) identifies two types of BR CUEs: compact type (solid and ball-like structures denoted by four arrows in upper figure); diffuse type (much larger, extended structure denoted by an arrow in the upper middle of the figure). The intensity of the staining is much higher in the former. Both types share the same myelinated afferent (green axons). An arrowhead denotes the diffuse type, which is much more observable in the bottom black and white figure, where two arrows denote two other smaller diffuse CUEs. The scale bar is 100 μm [Figure 3 (Liu et al., 2021)].

A recent study provides morphologic and physiologic data supporting MST in the BR unit (Liu et al., 2021). APs are generated at the first or second Ranvier node where sodium channels are highest in density. Using histochemical staining of the myelin sheath, the AP generating site can be identified at the demyelination junction point (Liu et al., 2021). Each CUE is clearly a sensor, not a contributor (Figure 8), because each is supplied with a myelinated axon. Several compact CUEs share an axon with a diffuse CUE, supporting the heterogeneous scenario (Figure 9). Thus, compact CUEs may represent rapidly adapting BRs (RBRs) and diffuse CUEs represent slowly adapting BRs (SBRs) or *vice versa*.

**FIGURE 10:**
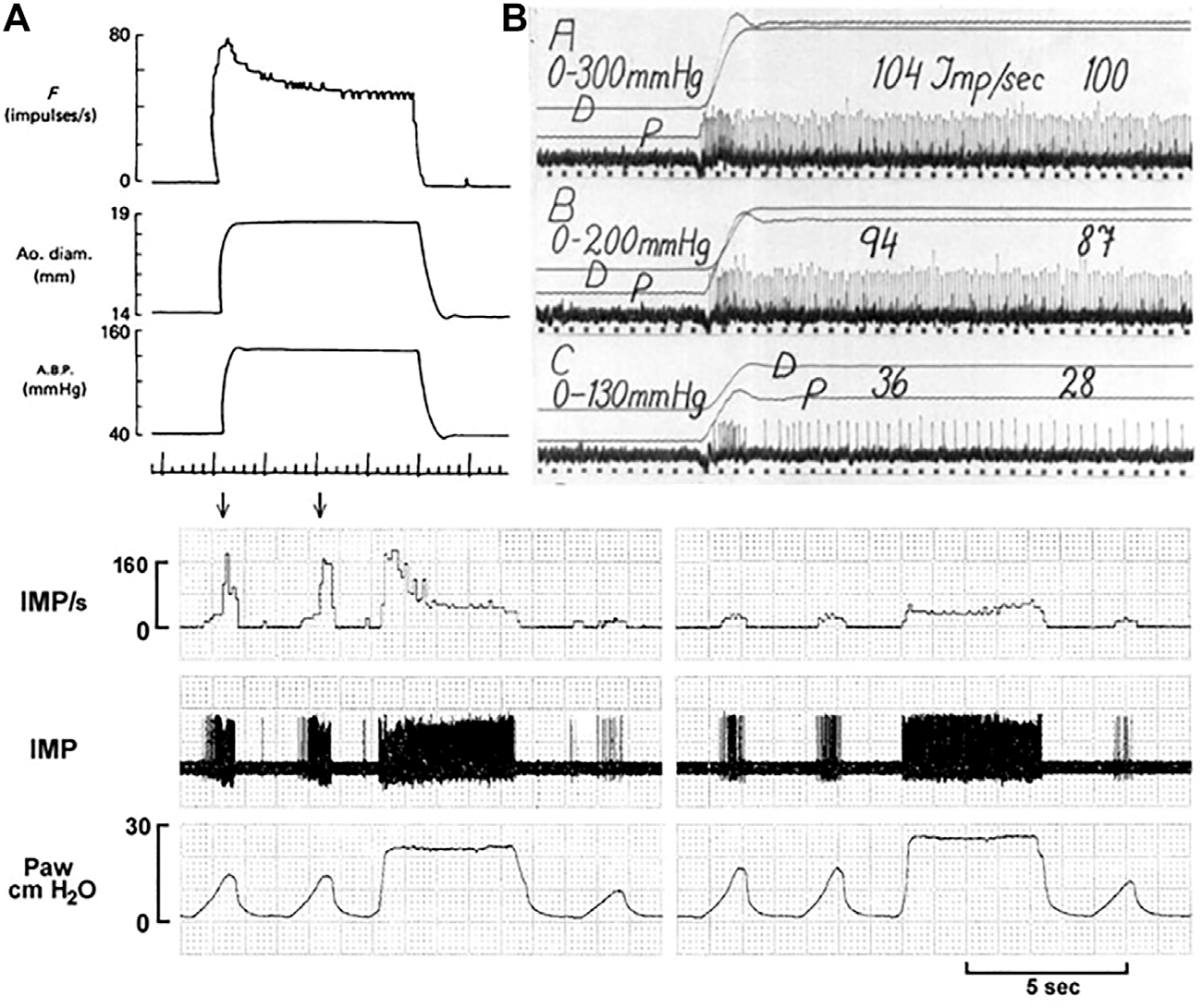
Unit adaptation in both processed data (left) and original recording (right). Left: A dog aortic BR unit in response to a step increase in constant aortic pressure. Traces: baroreceptor impulse frequency (F), aortic diameter, aortic pressure and time trace (1 s). [Figure 8 (Coleridge et al., 1984)]. Right: A cat carotid sinus BR unit in response to different distending pressure steps from 0 to 130, 200 and 300 mmHg. *p*, Intrasinus blood pressure; D, Diameter of the sinus. The time marks are 1/25 s [Figure 9 (Landgren, 1952a)]. Bottom. This airway unit contains RAR (high threshold and frequency) and SAR (low threshold and frequency) sharing an afferent axon. In **(A)**, during lung inflation the unit starts with SAR activation, producing a low discharge frequency; as the airway pressure increases, the RAR activates (denoted by two arrows), and discharges with very high frequency. During constant lung inflation the unit initially discharges with a higher RAR frequency, but rapidly adapts to a SAR steady state. In **(B)**, two receptive fields are identified connected to the afferent. Blocking one field with 2% lidocaine, the rapidly adapting component disappeared and left only the slowly adapting component, which exhibited continuous lower discharge frequency. IMP, impulses; Paw, airway pressure. [Figure 6 (Liu and Yu, 2013)].

Under MST, RBRs sense rate of change during pulsatile perfusion and discharge during the systolic phase. SBRs sense magnitude of change and usually discharge during both the systolic and diastolic phases. Most BR units are heterogenous, containing RBRs and SBRs. During pressure step, they present an initial rapidly-adapting component, and a later slowly-adapting component (Figure 10). There is a clear separation of the rapid and slow components in Figure 10 top right C. In lung units these rapid and slow components can be seperately blocked by local injection of anesthetics (Figure 10 bottom part). With this direct injection technique, different types of lung sensors are found to share an axon (Walker and Yu, 2021). However, this cannot be applied in CV units because the receptive fields cluster in a small region, unlike in the lung where they may extend a few centimeters apart.

**FIGURE 11:**
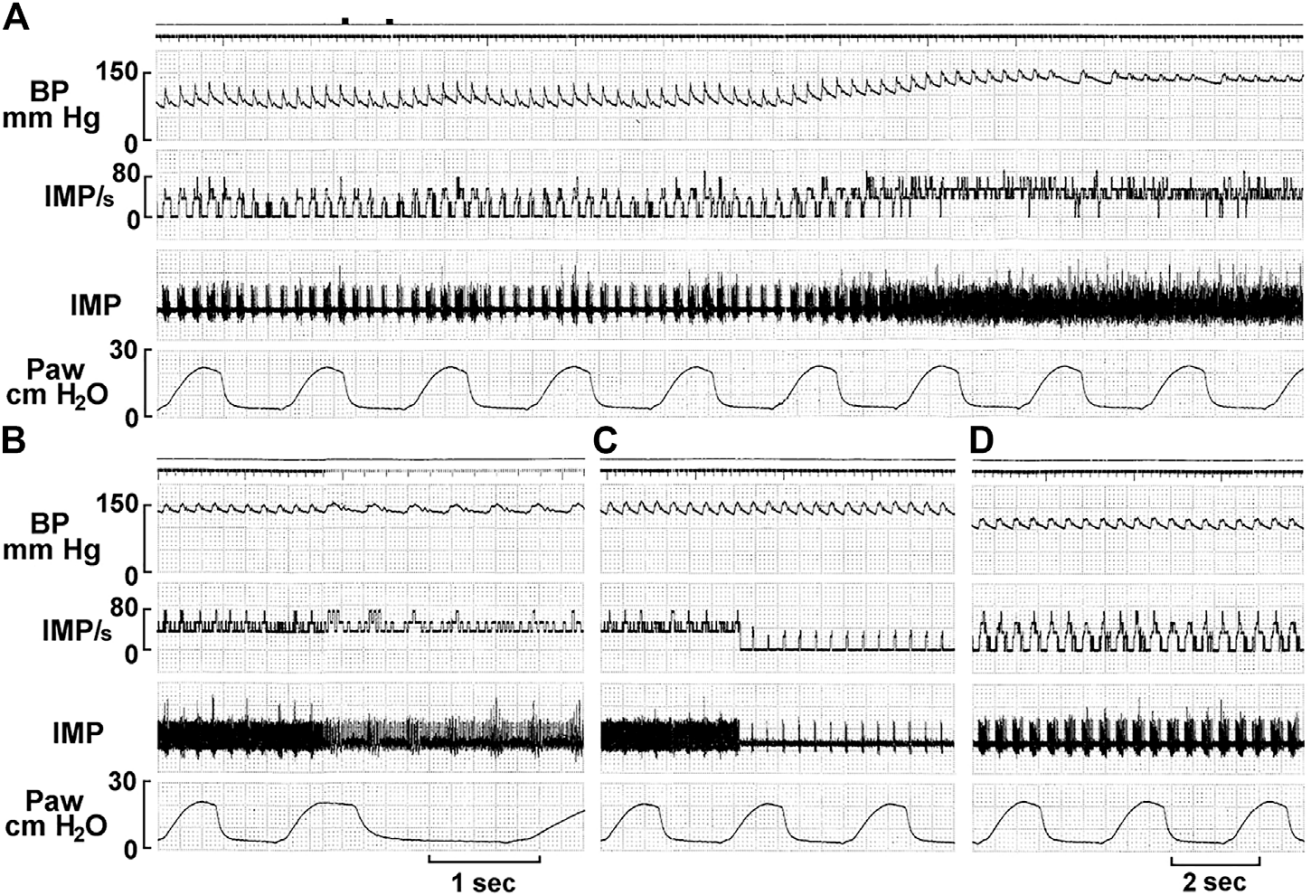
A single unit possesses multiple baroreceptors. **(A)** Unit activity of aortic baroreceptors recorded from the depressor nerve of a rabbit. The traces from top to bottom are: arterial blood pressure, impulse activity per second, impulse activity, and airway pressure. The time elapsed between **(A–D)** were 50, 50, and 430 s, respectively. The two black markers on the top of A indicate injection of a vasopressor (phenylephrine, 10 mM, 0.2 ml). Unit activity increased as BP increased **(A)**, and discharged continuously at higher frequency for 129 s and then abruptly decreased **(C)**, with activities in phase with cardiac rhythm. Please note that the discharge frequency is low even though BP stays high at 150 mmHg, which is due to deactivation, causing a pacemaker switch. The discharge pattern returned to normal 420 s after the deactivation **(D)** as the blood pressure became normal. [Figure 8 (Liu et al., 2021)]. That is, activity increases as the BP decreases, which against the doctrine that BRs are stimulated by increased pressure. This is strong evidence of MST.

MST is further supported by BR deactivation studies (Figure 11). In some units exposed to sustained pressures, activity decreases or completely shuts-off. This phenomenon is termed deactivation and probably results from overexcitation-induced energy supply shortage (Guardiola et al., 2007). For example, injecting saline or blood into sheep carotid sinus may increase activity in some BR units initially, followed by total deactivation. This phenomenon can be reproduced by withdrawing and reinjecting blood (Biscoe et al., 1969).

On close examination of the literature, different investigators have observed CV unit deactivation in response to over-stretch. The stimulus can be pressure (Arndt, 1979; Gilmore, 1979) or dp/dt (Mifflin and Kunze, 1984) (Table 1). As stretch increases, sensor discharge ensues until a point when activity decreases to a low level in the face of increased stretch. This indicates multiple sensor behavior. After the highest sensor deactivates, the next highest sensor becomes the pacemaker; when the second deactivates, the third takes over, and so on. Therefore, deactivation could be a series of events [Figure 8 of Ref (Angell James, 1971) and Figure 10 of Ref (Liu et al., 2021)].

**TABLE 1:** Mechanosensory deactivation.

Type Units	Species	Stimulus	Figure	References
Carotid Sinus	Sheep	Pressure	4	Biscoe et al. (1969)
	Cat		7	Landgren (1952a)
			2,3,5,9	Landgren (1952b)
Aorta	Cat	Pressure	6	Ninomiya and Irisawa (1967)
	Rabbit		8	Angell James (1971)
	Dog		4	Coleridge et al. (1981)
Atrium	Dog	Pressure	5.7	Arndt (1979)
			7.7, 7.8	Gilmore (1979)
			14	Gilmore and Zucker (1974)
	Cat	Balloon inflation	6,7	Chapman and Pankhurst (1976)
	Rat	dp/dt; Pressure	5,6; 6	Mifflin and Kunze (1984)


The evidence presented thus far strongly suggests MST operates across cardiopulmonary system organs. By extension, MST may inform several previously unexplainable issues.

### Multiple sensor theory interpretation

**FIGURE 12:**
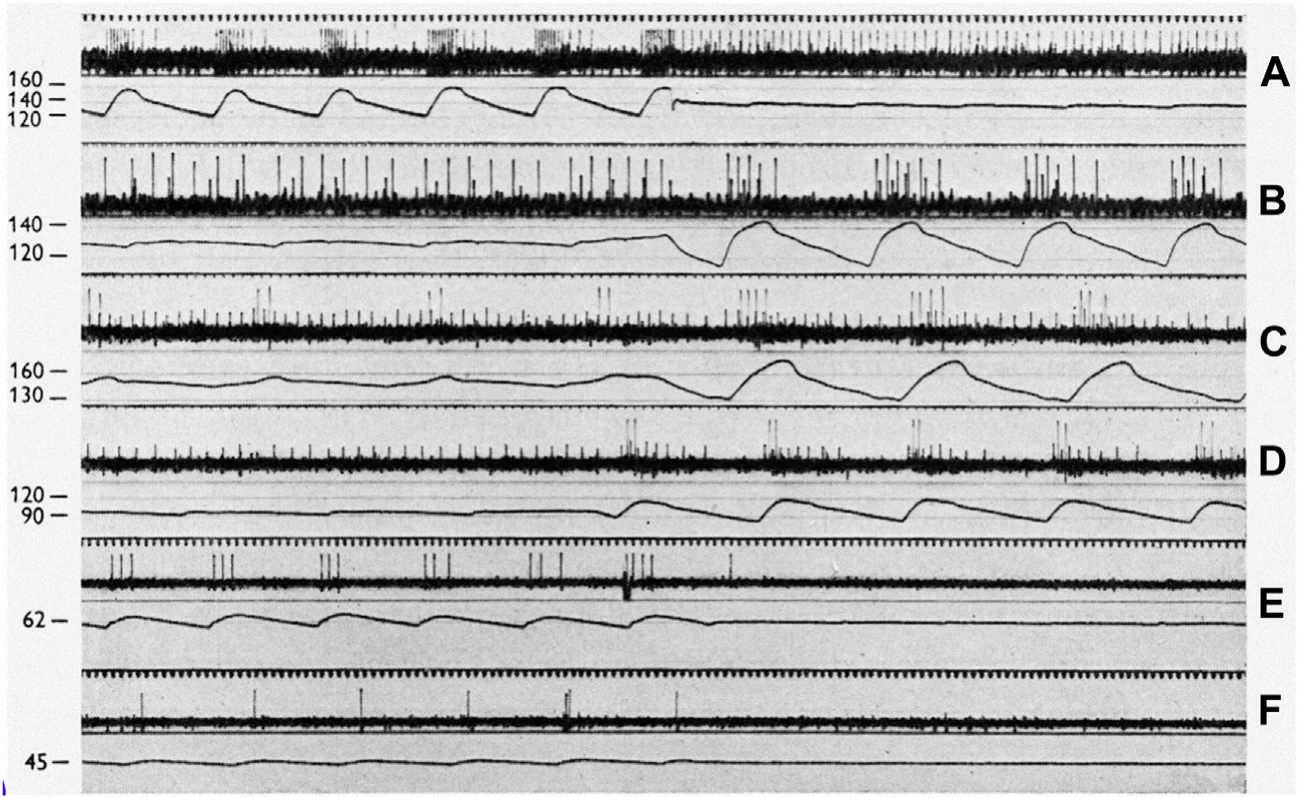
On a 50 cycles/sec time scale, each panel displays sinus baroreceptor unit activity in response to sinus pressure (SP) labeled in mmHg to the left. **(A)**: effect of transition from pulsatile to non-pulsatile sinus blood flow at SP of 140 mmHg (cat l). **(B)**: transition from non-pulsatile to pulsatile state at SP of 130 mm Hg (cat 2). **(C,D)** (cat 3) transitions at 150 and 95 mmHg, respectively. Please note that the small action potentials are from a SBR and the large ones from a RBR unit. **(E,F)** (cat 4) transitions at 62 and 45 mmHg, respectively. [Figure 1 (Ead et al., 1952)].

**FIGURE 13:**
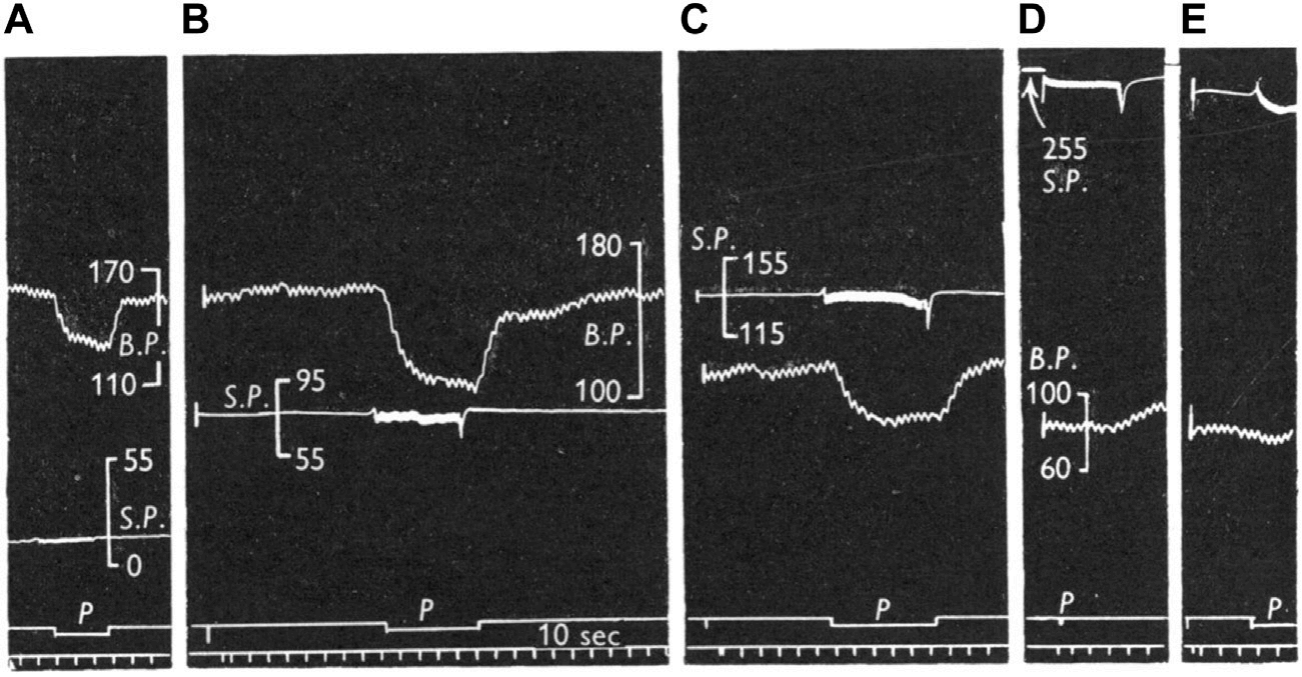
Comparison of reflex effects of pulsatile and non-pulsatile carotid sinus perfusion at mean pressures of 15 **(A)**, 80 **(B)**, 135 **(C)**, 240 **(D)**, and 235 **(E)** mmHg in the cat. Traces: BP, systemic blood pressure; SP, sinus perfusion pressure; *p*, indicates pulsatile; signal marker, time in I0 sec intervals. [Figure 3 (Ead et al., 1952)].

This section considers examples of MST’s utility in data interpretation and approaches several controversial issues. In Ead et al. (1952) systematically studied BR behavior and reflex responses. Their key recordings (Figure 12 and Figure 13) nicely demonstrate MST.

Figures 12A,B demonstrate typical heterogenous BR units. In A, RBR discharge is seen on the upstroke phase during pulsatile systolic pressure, which converts to sporadic SBR APs. SBR behavior can be verified by non-adapting discharge during non-pulsatile flow.

In C and D, the small and large APs display SBRs and RBRs, respectively. The SBR unit is nearly saturated at non-pulsatile 150 mmHg because the frequency is about the same at 175 mmHg (pulsatile peak, C). Its discharge becomes cyclic at 95 mmHg pulsatile perfusion (D). On close inspection of the pulsatile cycle, activation thresholds are the same during systole and diastole (slowly adapting behavior). In addition, they discharge non-adaptively when pressure pulse is significantly reduced (D). In contrast, the RBR unit activates only during the systole with large pulse pressure (high dp/dt) at 95 mmHg (D), generating higher instantaneous frequency than the small pulse pressure at 150 mmHg (C), because the two APs are closer together in D. In C, minimal pressure during the reduced pulsation is 140 mmHg, which is significantly higher than the pulsatile peak pressure (120 mmHg) in D. If this were SBR, the discharge frequency should be higher in C than D.

12E and F also illustrate RBR behavior because the unit activates during systole. If it were SBR it would discharge more during the non-pulsatile phase in E than the pulsatile phase in F.

From the foregoing, there are three types of sensory behavior: RBR, SBR, and heterogenous. These correspond with bronchopulmonary RARs, SARs, and intermediate receptors (Yu, 2020). However, in the CV system, RBRs usually have a lower threshold than SBRs, as illustrated in Figure 10 and Figure 12. Lower threshold RBRs may share an afferent axon with high threshold SBRs [Figures 3, 5, 9,14, and 18 of Ref (Landgren, 1952b)]. Thresholds low in RBRs and high in SBRs are the key property of BR units. In the lung, it is reversed: thresholds are usually higher in RARs than SARs. Thus, as constant pressure increases, the rapidly adapting component usually decreases in BR units (Figure 10 and Figure 11), but increases in lung units (Yu, 2020).

The above interpretation of how of sinus pressure (SP) influences RBR/SBR behavior provides a sensory basis to interpret the effects of SP on blood pressure under pulsatile and non-pulsatile conditions. In Figure 13 the right carotid sinus was isolated and perfused, with the left sinus and both aortic nerves cut. At 15 mmHg non-pulsatile SP (A), BP is about 160 mmHg and represents the highest level during rest with no buffering effect from BR input because there is no RBR and SBR activity. Even though some RBRs reached their activation threshold, their activity adapts during the non-pulsatile phase and SBR threshold is usually about 80 mmHg. Shifting perfusion from non-pulsatile to pulsatile activates low-threshold RBRs. Therefore, BP drops from 160 to 130 mmHg. Returning to non-pulsatile perfusion eliminates RBR activity; therefore, BP returns back to 160 mmHg (A). In B, at 80 mmHg non-pulsatile SP, the BP is also 160 mmHg because, similar to A, there is no BR activity. However, once perfusion becomes pulsatile, BP dramatically drops to 110 mmHg as many more RBRs are activated. In C, at SP perfusion of 135 mmHg, many SBRs are activated. Therefore, baseline BP decreases to 115 mmHg. Switching to pulsatile perfusion, BP drops to 88 mmHg. At this time, BP drop is less than that in B, because RBR activities generated at this perfusion level are lower. Finally, at SP of 240 (D) and 235 (E) mmHg, BP decreases to the lowest level (83 mmHg) due to maximum activation of BR units. In other words, at these extremely high SP levels, RBRs and SBRs are fully saturated and no further buffering effects on BP will occur.

Considered next are several unresolved mechanosensory issues and how they might be approached under MST.

1) Pacemaker switching from one sensor to another is unexplainable sensory behavior. To illustrate, excerpts from Angell’s 50-year-old report on BR behavior are interpreted under MST (in parentheses). “At a certain pressure the relationship between the impulse frequency and intra-aortic pressure ceased to be linear and this pressure is described as the point of inflexion. The impulse frequency at the point of inflexion was not necessarily the maximum frequency attained in any given fibre (switching to another sensor with its own properties); in some fibres, at least, the impulse frequency continued to increase with pressure (switching to an unsaturated sensor) whereas in others the impulse frequency either remained constant (switching to a sensor that saturated at the pressure level), diminished (switching to an unsaturated sensor with less sensitivity), became intermittent (during a process of a series of deactivation) or decreased to zero (complete deactivation).” (Angell James, 1971). Relatedly, “we have occasionally observed one and the same ending give first one type of response and then the other” (pacemaker switch) (Bronk and Stella, 1932). Pacemaker switch can also explain resetting. In Figure 11C the unit switches from continuous pattern to pulsatile pattern, i.e., switching from SBRs to RBRs. These observations are typical of pacemaker switching. These phenomena are also observed in lung sensors. For example, unit activity switches from occurring in inflation to deflation (Yu, 2022).
2) Resetting mechanism. Resetting is defined as a shift of the stimulus-response curve. It divides into instantaneous, acute (or rapid), and chronic types. Instantaneous resetting presents in a cardiac cycle: the BR is inactive at much higher pressures in diastole than in systole. Acute resetting occurs in seconds or minutes after a sustained change in arterial pressure. Chronic resetting takes days to years to complete, which may involve vascular structural changes (Chapleau et al., 1991). In OST, mechanisms for instantaneous and acute resetting are believed to be mechanical changes in the vessel wall and ionic factors involving the electrogenic sodium pump in the sensor (Chapleau et al., 1991; Koushanpour, 1991). In MST, instantaneous resetting is due to activation of both RBRs and SBRs in a heterogeneous unit (Figure 12A). Mechanosensor deactivation contributes to acute resetting (Figure 11). While MST does not exclude potential mechanical and ionic mechanisms, deactivation causing decreased discharge frequency is obvious (Figure 11). Mechanical and ionic theories cannot explain sudden decrease in BR activities (deactivation) and continuous discharge pattern shifts to a cyclic pattern after deactivation (Figure 11). Deactivation also occurs in atrial receptors (Table 1) and it may explain that maximal discharge decreases after 15 min of distension without any mechanical change in rat superior vena cava (Mifflin and Kunze, 1982), supporting resetting occurs in the sensory unit.
3) Adaptation mechanism. It has been observed, “the activity of single baroreceptor fibres increased abruptly with the rise in pressure, decreased rapidly at first, then gradually, and often ceased abruptly despite the maintained increase in pressure” (Chapleau et al., 1993). And 4-AP did not alter peak discharge frequency, but significantly attenuated its decline and prevented or delayed abrupt cessation of activity. Thus, 4-AP was claimed to decrease sensor adaptation rate. However, MST interprets this differently. The initial rapid component is from RBRs and sustained activity from SBRs. Abrupt cessation results from complete deactivation. 4-AP increases SBR basal activity, decreases its deactivation, and has no effect on RBR adaptation, because the RBRs generate the same peak frequency and adapt at the same rate, but to a higher SBR discharge level [Figures 1, 4 (Chapleau et al., 1993)]. This gives a lower adaptation rate of the unit, but does not affect sensor adaptation rate at all.
4) Effects of pulsatile pressure on BR activity. At a given static pressure, superimposed pulse pressure potentiates BR activity. This interaction is especially prominent at the lower pressure range with higher pulse pressure [Figure 2 (Koushanpour, 1991)]. However, at high static pressure, superimposed pulse pressure shows little effect on BR activity. This phenomenon can be explained by MST, since RBRs have a lower threshold than SBRs. RBRs activate at low pressure pulsation, causing significant dynamic component. At very high pressure, activity mainly comes from SBRs, because SBR activity (having higher discharge frequency) overrides RBR activity. When the unit activity is saturated, pulsation can generate no further dynamic activity (Figures 13D,E). This also explains how pulsatile pressure can prevent acute BR resetting caused by non-pulsatile perfusion, which is not explained very well under OST (Mendelowitz and Scher, 1990; Koushanpour, 1991). Under MST, static stimulation activates SBRs continuously, causing high discharge frequency leading to deactivation. Adding pulsatile pressure, RBRs activate first and then shift to SBRs. In the interaction process the SBR rests periodically, therefore decreasing deactivation, i.e., resetting (Mendelowitz and Scher, 1990). The involvement of RBRs also explains why greater pulsatile stimulus (higher dp/dt) is more effective in preventing resetting. This is because more RBRs activate, taking on a larger share of the workload. Thus, high pulse pressure and low perfusion pressure are more effective to prevent resetting. This may explain some differences in effectiveness of pulsatile pressure in prevention of resetting reported by different investigators.
5) Conversion of atrial receptor pattern. Under MST, Types A and B atrial receptors respond to atrial contraction and filling, respectively. This indicates they are different types of sensors operating with different mechanisms. Intermediate receptor behavior results from combined activation of Type A and B in the unit. Looking at Figure 5, although infusion did not change a-burst discharge pattern significantly, there is clear interaction between the v-burst and a-burst. A complete conversion in MST is due to activation of one type at one status and activation of another type at another (Figure 6). To accept MST, a legitimate question is how the CNS decodes incoming signals from different sensor types lying within the unit. Many mechanisms are possible. For example, an atrial unit may carry both A and B signals, providing precise high discharge frequency at each atrial contraction, and gradual increase in activity during the filling phase. These events occur in different time domains and their pattern can be easily deciphered. Such potential decoding mechanisms have been discussed in the respiratory sensory unit (Yu, 2005; Yu, 2020; Yu, 2022).

### Critiques of multiple sensor theory

This section discusses several reviewer critiques of MST.

1) Action potential wave forms on the time scales presented in most of the figures are easy to overinterpret, without some other corroborating approach to state that there really is only 1 unit in the recording, i.e., conduction velocity analysis.

Response: Regarding single unit evidence and risk of overinterpretation, single unit recordings in each figure cited were verified by action potential waveforms under an oscilloscope. Action potential waveforms are the basis for identifying a single unit. Using conduction velocity to further corroborate single unit recordings is a great suggestion. However, it nearly impossible using currently available techniques. Sensory axons converge at multiple levels in the airway and lung. Daughter axons cannot be identified for conduction velocity assessment: there is too little space to operate within the airway. This problem is even more prominent in CV baroreceptor recordings because the sensors cluster even closer together. Still, in my view, within an action potential interpretive scheme, ample literature-wide evidence supports within-unit heterogeneous mechanosensor behavior along single axons. Since conduction velocity is related to axon size, morphological studies can be used to indirectly assess the conduction velocity by measuring axon diameter of different sensors. Please see critique 2.

2) One argument against MST would be if the SAR and RAR were conducted along axons with different conduction velocity. There is significant differences between SAR and RAR conduction velocities in the lungs (although the overlap is substantial). What is known of the conduction velocity of type A and B in the atria, and of slowly and rapidly adapting baroreceptors? And how can these different conduction velocities be incorporated into MST?

Response: This argument points out a potentially difficult problem under conventional OST interpretation. That is, in order to verify MST, differences in conduction velocity of RARs and SARs in a single unit need to be demonstrated. In addition, after convergence of slow RARs and fast SARs, what is the conduction velocity of the unit? However, the interpretation scheme is completely different under MST. Indeed, claims of different conduction velocity underpin OST as a way to demonstrate that RARs and SARs are different type of sensors or units. Yet no single study so far convincingly supports this claim. On the contrary, four of the most credible studies show RAR and SAR conduction velocities completely overlap [see answer to question three in the Ref (Yu, 2020)]. In 3 cases, they are superimposed at the lower end of SAR conduction range. In the other case, they are exactly the same. Thus, RARs and SARs have the same conduction velocity. The existence of some fast conduction SAR units cannot be explained by OST but can be potentially explained by MST due to unit selection criteria and has been dealt with in detail (Yu, 2020). In early studies, scientists believe that RARs and SARs can be selectively blocked by cooling the temperature from vagus nerve, due to their difference in axon diameters. However, this type of study turned out to be nonproductive.

Regarding conduction velocity in type A and B atrial receptors, both are myelinated afferents with significantly overlapping conduction velocities (Paintal, 1973). The literature provides little further information. Notably, Paintal and many other investigators strongly believe types A and B are different sensors. In my view, if types A and B showed reliably different conduction velocities, such reports would be a point of emphasis in support of the two different types. Moreover, the available morphologic information does not demonstrate differences in A and B axon thickness (Figure 7B) if you believe type A and B are associated with intramuscular and flower-spray endings, respectively.

Regarding slowly and rapidly adapting BRs, this review outlines my interpretation. In general, OST describes the BR as slowly adapting with dynamic properties. My view is rapidly and slowly adapting components operate on the BR unit (Figure 10) and derive from RBRs and SBRs, respectively. So far no one else refers to different types of BRs, although RBRs, SBRs and mixed RBRs with SBRs are demonstrated (Figures 10–12). As is in pulmonary and atrial mechanosensors, there is no information on differences in conduction velocity between RBRs and SBRs. Similarly, the available morphologic information indicates no differences if you believe compact and diffuse CUEs represent RBRs and SBRs or *vice versa* (Figure 9).

The next issue is how to take into account RAR/SAR conduction velocities in MST? RAR and SAR conduction velocities are assumed to be different within an OST framework. Therefore, RARs travel in small axons and SARs in large axons going to different parts of CNS. Yet there is no evidence or reasoning behind RARs being smaller than SARs. RARs and SARs should terminate the same sites in the CNS [see section of Central Projection in a book chapter (Yu, 2022)]. The smallest and largest SARs are the same type of sensors in behavior and function and so are the smallest and largest RARs. Axon diameter does not affect sensory behavior nor should sensory behavior influence axon diameter. I believe RAR and SAR conduction velocities are the same. Conduction velocity itself does not inform any sensory property. The myelinated axons just provide fast conduction for action potentials. It is discharge patterns of action potentials that provide the information. Therefore, the velocity arguments neither support nor discredit MST. Whether or not RARs and SARs have different conduction velocities would have no apparent bearing on MST.

3) Deactivation/desensitization does not *per se* mean multiple sensors are expressed by the same unit.

Response: I agree deactivation is sensory behavior. The sensor stops activity when over-excited; however, at any instant the active sensor is the pacemaker. Unit activity drops to a new pacemaker with less discharge if the active sensor deactivates. This phenomenon can be used to detect multiple sensors as we demonstrated in Figure 11, where the activity drop is sudden, which is followed by a regular cyclic discharge. These two different levels of activity are best explained by MST.

4) Figure 8: “Each CUE is clearly a sensor, not a contributor”. It is unclear how an image of the arbor of the afferent can definitely explain the functional contribution of each of its terminations.

Response: It is important to look at morphology and physiology together. I had the same difficulty in identifying a sensory structure as a sensor or contributor before myelin basic protein (MBP) staining approach (Yu, 2005). MBP labels myelinated axons, whose sheaths on morphology terminate just before the CUE (Figure 8 and Figure 9), indicating that first or second node is located at this point. Since action potentials are generated at the nodes, each CUE can generate action potentials and are considered as a sensor or encoder.

## Conclusion

Strong evidence points to MST operating on CV BR behavior in much the same manner well-established in the lung mechanosensors. Under MST, the sensory unit carries out two major functions: 1) mechano-electrical transduction, which converts mechanical forces into electrical signals; and 2) sensory integration, which processes electrical activity from all heterogeneous sensors in the unit. This conceptual change requires data reinterpretation and alters future research design. Understanding unit encoding forms a basis to delineate CNS decoding operation (likely a reverse process). Moreover, understanding peripheral sensory integration may inform the study of CNS integration, because common mechanisms are likely conserved.
